# Pendrin abundance, subcellular distribution, and function are unaffected by either αENaC gene ablation or by increasing ENaC channel activity

**DOI:** 10.1007/s00424-023-02797-w

**Published:** 2023-03-29

**Authors:** Johannes Loffing, Vladimir Pech, Dominique Loffing-Cueni, Delaney C. Abood, Young Hee Kim, Chao Chen, Truyen D. Pham, Jill W. Verlander, Susan M. Wall

**Affiliations:** 1 Institute of Anatomy, University of Zurich, Zurich, Switzerland; 2 Division of Renal Medicine, Department of Medicine, Emory University, Atlanta, GA 30322 USA; 3 The Division of Nephrology, Hypertension and Renal Transplantation, The University of Florida College of Medicine, Gainesville, FL USA

**Keywords:** Pendrin, ENaC, Aldosterone, Intercalated cells

## Abstract

The intercalated cell Cl^−^/HCO_3_^−^ exchanger, pendrin, modulates ENaC subunit abundance and function. Whether ENaC modulates pendrin abundance and function is however unknown. Because αENaC mRNA has been detected in pendrin-positive intercalated cells, we hypothesized that ENaC, or more specifically the αENaC subunit, modulates intercalated cell function. The purpose of this study was therefore to determine if αENaC is expressed at the protein level in pendrin-positive intercalated cells and to determine if αENaC gene ablation or constitutively upregulating ENaC activity changes pendrin abundance, subcellular distribution, and/or function. We observed diffuse, cytoplasmic αENaC label in pendrin-positive intercalated cells from both mice and rats, with much lower label intensity in pendrin-negative, type A intercalated cells. However, while αENaC gene ablation within principal and intercalated cells of the CCD reduced Cl^−^ absorption, it did not change pendrin abundance or subcellular distribution in aldosterone-treated mice. Further experiments used a mouse model of Liddle’s syndrome to explore the effect of increasing ENaC channel activity on pendrin abundance and function. The Liddle’s variant did not increase either total or apical plasma membrane pendrin abundance in aldosterone-treated or in NaCl-restricted mice. Similarly, while the Liddle’s mutation increased total Cl^−^ absorption in CCDs from aldosterone-treated mice, it did not significantly affect the change in Cl^−^ absorption seen with pendrin gene ablation. We conclude that in rats and mice, αENaC localizes to pendrin-positive ICs where its physiological role remains to be determined. While pendrin modulates ENaC abundance, subcellular distribution, and function, ENaC does not have a similar effect on pendrin.

## Introduction

Within the mammalian kidney, the connecting tubule (CNT) and collecting duct (CCD) are made up of intercalated cells, principal cells, and connecting tubule cells [46]. Intercalated cells (ICs) have 3 subtypes, type A, type B and non-A, non-B intercalated cells. The Cl^−^/HCO_3_^−^ exchanger, pendrin, localizes to the apical regions of both type B and non-A, non-B intercalated cells [46]. These pendrin-positive intercalated cells mediate Cl^−^ absorption and HCO_3_^−^ secretion, particularly in models of metabolic alkalosis [46]. Type A intercalated cells secrete H^+^’s, most notibly in models of metabolic acidosis and do not express pendrin [46]. Principal cells within the CCD and connecting tubule cells in the CNT mediate Na^+^ absorption primarily through the action of the epithelial Na^+^ channel, ENaC, which plays a vital role in NaCl balance and therefore blood pressure regulation [10, 30]. However, pendrin-positive intercalated cells are also important in Na^+^ [15, 18] and Cl^−^ [41, 45] balance, as well as in blood pressure regulation [39, 45] by mediating Cl^−^ absorption, and by modulating ENaC-mediated Na^+^ absorption [15, 24, 27].

The nature of the interaction between pendrin and ENaC is not fully understood. While there is abundant evidence that pendrin modulates ENaC abundance and function [15, 24, 27, 35], whether ENaC modulates pendrin abundance and/or function is unknown. Initial studies observed α, β, and γENaC subunit expression within principal cells and connecting tubule cells, but not intercalated cells [12]. However, more recent RNAseq studies detected high levels of αENaC transcript (*Scnn1a*) in pendrin-positive intercalated cells of the mouse, although β and γENaC transcripts were not detected in that cell type [4]. Moreover, our previous work showed that Cl^−^ absorption and transepithelial voltage are much lower in CCDs from aldosterone-treated mice that are αENaC null within both intercalated cells and in principal cells than in floxed αENaC controls [26]. Because pendrin is critical to Cl^−^ absorption in CCDs from both aldosterone-treated and NaCl-restricted mice [39, 45], we asked if ENaC modulates pendrin-dependent Cl^−^ absorption in mouse models associated with high circulating aldosterone.

Whether αENaC protein localizes to mouse intercalated cells, and whether this subunit, or the ENaC channel more broadly, modulates intercalated cell transporter abundance and/or function has not been established. The purpose of this study was therefore to determine if αENaC subunit protein is expressed within ICs, to determine the subcellular distribution of αENaC within these cells and to determine if αENaC gene ablation or increasing ENaC channel activity change pendrin abundance, subcellular distribution, or function.

## Methods

### Animals

We studied mouse models of Pendred Syndrome (*Slc26a4 *^(–/–)^ or Pds^–/–^) [7] and Liddle’s Syndrome (LL) [30] that have been described previously. Liddle’s mice and their wild type littermates used in immunogold experiments were on a C57Bl6/J background. Homozygous pendrin null (*Slc26a4*^−/−^) and mouse models of the Liddle’s mutation (ENaC^LL^), both on a 129S6SvEvTac background, were bred to produce littermates that were (1) homozygous pendrin null and homozygous for the Liddle’s mutation (ENaC^LL^; *Slc26a4*^−/−^ or LL/KO), (2) homozygous for the Liddle’s mutation and homozygous wild type pendrin (ENaC^LL^; *Slc26a4* ^+/+^ or LL/WT), (3) homozygous pendrin null with wild type ENaC (ENaC^WTWT^; *Slc26a4*^*−/−*^ or WT/KO), and (4) mice homozygous for both wild type pendrin and ENaC (ENaC^WTWT^; *Slc26a4*^+*/*+^ or WT/WT). These mice have been described previously [25, 27]. Other experiments compared collecting duct-specific αENaC null mice (HoxB7:Cre *Scnn1a*^loxloxcre^) with control (*Scnn1a*^loxlox^) littermates on a C57Bl6/J background, which have been described previously [33]. Mouse genotype was determined from tail biopsies by PCR with specific probes designed for each gene (Transnetyx, Cordova, TN). In other experiments, we used archived paraformaldehyde-fixed kidney tissue from male, wild type C57Bl6/J mice and Wistar rats that were 2–4 months of age.

### Mouse conditioning

#### Treatment #1: a standard NaCl diet

Mice ate a standard, rodent diet (KLIBA NAFAG #3430, Kaiseraugst, Switzerland) and drank water ad libitum.

#### Treatment #2: aldosterone and a NaCl-replete diet

For 5–10 days prior to sacrifice, mice ate a balanced diet (53881300; Zeigler Brothers) prepared as a gel (0.6% agar, 74.6% water, and 24.8% mouse chow) supplemented with NaCl (~ 0.8 mEq NaCl and 0.8 mEq K^+^ per day) [15] and received aldosterone by minipump (250 µg/kg body weight/day).

#### Treatment #3: NaCl-restricted diet

For 7 days, mice ate the gelled diet described in Treatment #2, but without the added NaCl and without aldosterone administration. Thus, mice ingested 0.13 mEq NaCl and 0.8 mEq K^+^ daily [44].

Each day mice were given a cup with a fixed quantity of gel by weight. That cup was removed 1 day later, weighed to determine the amount of gel consumed over the previous day, and then replaced with another cup. That process was repeated until the end of the protocol. The Institutional Animal Care and Use Committee at Emory University and the Veterinary Office of the Canton of Zurich approved all treatment protocols.

### Measurement of net transepithelial Cl^−^ flux in CCDs perfused in vitro

CCDs were dissected from medullary rays at 11 °C in HCO_3_^−^-buffered physiological solution containing in mM: 125 NaCl, 24 NaHCO_3_, 2.5 K_2_HPO_4_, 2 CaCl_2_, 1.2 MgSO_4_, and 5.5 glucose, equilibrated with 95% air/5% CO_2_, as described previously [32]. Tubules were perfused and bathed in the same solution at flow rates of 2–3 nl/min. CCDs were equilibrated at 37 °C for 30 min prior to starting the collections.

Cl^−^ concentration was measured in perfusate and collected samples using a continuous-flow fluorimeter and the Cl^−^ sensitive fluorophore, 6 methoxy-N-(3-sulfopropyl) quinolinium (SPQ; Molecular Probes, Eugene, OR), as described previously [9, 42]. Based on the Cl^−^ concentration measured in the collected samples and the flow rate, transepithelial Cl^−^ flux was calculated as described previously [42].

### Immunogold cytochemistry and morphometric analysis

Pendrin was localized in ultrathin sections using immunogold cytochemistry as described previously [43]. Intercalated cell subtypes were identified based on the morphological characteristics of mouse intercalated cell subtypes established previously [43]. Apical plasma membrane boundary length, cytoplasmic area, and gold label on the apical plasma membrane and in the cytoplasm were quantified in type B and non-A, non-B intercalated cells [39, 43]. For each intercalated cell subtype, at least five cells were selected from each mouse at random and photographed at a primary magnification of × 5000 and then examined at a final magnification of approximately × 18,200. For each animal, raw morphometric data from each cell profile were pooled to obtain an average value. The “*n*” stated reflects the number of animals studied.

### Immunoperoxidase staining and quantitative analysis of immunohistochemistry

The kidneys were fixed in paraformaldehyde fixative in situ*,* as described previously [43] and embedded in paraffin or polyester wax [polyethylene glycol 400 distearate (Polysciences, Warrington, PA) and 10% 1-hexadecanol]. Two micron-thick sections were cut and mounted on gelatin-coated glass slides [22].

Immunohistochemistry used in montages was performed using standard immunoperoxidase procedures [16]. Endogenous peroxidase was blocked with 0.5% H_2_O_2_ in absolute methanol for 30 min at room temperature. For antigen retrieval, sections were incubated in 1 mM Tris solution (pH 9.0) supplemented with 0.5 mM EGTA and heated in a microwave oven for 10 min. Nonspecific binding of IgG was prevented by blocking in PBS supplemented with 1% BSA, 0.05% saponin, and 0.2% gelatin. Sections were incubated overnight at 4 °C with the anti-pendrin antibody [17], diluted in PBS supplemented with 0.1% BSA and 0.3% Triton X-100. Sections were rinsed with PBS supplemented with 0.1% BSA, 0.05% saponin, and 0.2% gelatin. Labeling was visualized with horseradish peroxidase-conjugated secondary antibody (1:200, DAKO), followed by incubation with 3,3′-diaminobenzidine (DAB; brown stain).

Sections used for quantitative immunohistochemistry were taken from the same tissue blocks as those above. Sections were dewaxed, rehydrated, and rinsed in distilled water. Endogenous peroxidase activity was blocked by incubating the sections for 45 min in 3% H_2_O_2_ in distilled water. Sections were then incubated with the primary anti-pendrin antibody at 4 °C overnight [17], diluted in Dako antibody diluent, washed in PBS and incubated for 45 min in polymer-linked peroxidase-conjugated anti-mouse IgG diluted to 1:10 or 30 min in anti-rabbit IgG (Vector ImmPRESS, Vector Laboratories, Burlingame, CA), washed again with PBS, and exposed to diaminobenzidine (Vector DAB substrate kit) for 5 min. Sections were then washed in distilled water, dehydrated in graded ethanols and xylene, mounted and examined with a Leica DM2000 microscope equipped with DIC optics and a Leica DFC425 digital camera and Leica DFC Twain Software and LAS application suite (Leica Microsystems, Buffalo Grove, IL), and observed by light microscopy.

Labeling was compared in sections of the same thickness from the same experiment using identical reagents. Two groups were examined when performed in the same immunohistochemistry experiment. Differences were evaluated by 2 blinded observers who agreed on the overall differences in label distribution and intensity between the 2 groups.

For quantification, high-resolution digital micrographs of defined tubule segments were taken in a random, systematic pattern using a Leica DM2000 microscope and a Leica DFC425 digital camera (14.4-megapixel images, 63X objective) and Leica DFC Twain Software and LAS application suite (Leica Microsystems, Buffalo Grove, IL). Some results were confirmed with images taken using a Leica DM 4500B microscope, a 63X objective, a Zeiss Axiocam 705 digital camera and with Zeiss Zen 3.4 software. Individual cells and their respective nuclei were circumscribed with ImageJ software (version 1.48v; National Institutes of Health). Net intensity at each pixel was determined as the difference between absolute pixel intensity and mean background intensity. Immunolabel intensity was quantified using custom-written software executed in Microsoft Excel 2010. Cell and nuclear area were determined as the number of pixels within the outlined region. Background intensity was determined in each photomicrograph by circumscribing cytoplasm in an unlabeled cell from the same tubule. For each cell measured, nuclear area and pixel intensity were subtracted from whole cell area and pixel intensity to determine cytoplasmic area and pixel intensity. We called this the “whole cell” method of quantifying pendrin label per cell. Values for pendrin label per cell reported herein employed this whole cell method. This total cell expression measurement was confirmed using a second method, which we call the “linear” method and involved integrating net pixel intensity across the entire cell, as described previously [40]. Immunoreactivity expressed at zones throughout the cell was determined by integrating pixel intensity at this region.

Transporter subcellular distribution was quantified in bright field light micrographs, as reported [40]. Pixel intensity across a line drawn from the tubule lumen through the center of an individual cell in high-resolution digital micrographs was quantified with NIH ImageJ, version 1.34 s software. Background pixel intensity was calculated as the mean pixel intensity outside the cell and was subtracted from the pixel intensity at each point.

Cell height was determined as the distance in pixels between the apical and the basolateral cell edges. Microscopy and data analysis were performed by an observer that was blinded as to the treatment group, genotype, and sex of each animal. Data from all pendrin-positive cells in the CCD or CNT were averaged for each animal and used in the statistical analysis.

### Immunnofluorescence

Consecutive sections of paraffin-embedded mouse kidneys were mounted on gelatin-coated glass slides and were deparaffinized in a graded series of alcohol and then rehydrated. For antigen retrieval, sections were boiled in 0.01 M Citrate Buffer (pH 6.0) for 10 min at 98 °C and then stored in ice-cold PBS for subsequent immunostaining. Consecutive, 3–4 mm cryosections were prepared from archived frozen mouse and rat kidneys (rat and mouse *Treatment #1*) and mounted on gelatin-coated glass slides. Unspecific binding sites in deparaffinized sections and in cryosections were then blocked for 30 min with 10% normal goat serum and sections were incubated at 4 °C for 12–16 h with the primary antibodies diluted in PBS/1%BSA. The primary antibodies used are the following: rabbit-anti-mouse αENaC [36] diluted 1/2500, chicken anti-H^+^-ATPase E subunit antibody [2, 23] diluted 1/500, mouse anti-bovine H^+^-ATPase [Mr = 31,000 subunit] [13] diluted 1/16, and rabbit-anti-mouse pendrin [11] diluted 1/10,000. Binding sites of the primary antibodies were revealed with red fluorescent Cy3-labelled goat-anti-rabbit IgG (Jackson ImmunoResearch; #111–165-144; diluted 1:1,000 in PBS/1%BSA), red fluorescent Alexa Fluor 555-labeled goat, anti-rabbit IgG (Invitrogen, #A32732, diluted 1:1000 in PBS/1% BSA), green-fluorescent Alexa Fluor 488-labelled goat-anti-chicken IgG (Invitrogen; #A11039, diluted 1:500 in PBS/1%BSA), and green fluorescent Alexa Fluor 488-labeled goat, anti-mouse IgG (Invitrogen, #A11029, diluted 1:200 in PBS/1% BSA). Sections were then rinsed with PBS. Coverslips were mounted using DAKO-Glycergel (Agilent Technologies, Santa Clara, CA), to which 2.5% 1,4-diazabicyclo[2,2,2]octane (DABCO; Sigma-Aldrich, St. Louis, MO) was added as a fading retardant. Sections were studied with a Leica Fluorescence Microscope (DM6000). Digital images were acquired with a charge-coupled-device camera and processed with Fiji (ImageJ) imaging software [34].

### Immunoblots

Immunoblotting used methods reported previously [16, 28]. Mouse kidneys were placed in an ice cooled buffer (0.3 M sucrose, 25 mM imidazole, pH 7.2, containing 1 × Roche Complete Protease Inhibitor Cocktail), immediately homogenized with an Omni THQ Tissue Homogenizer (Omni International) and then centrifuged at 1000 × g for 15 min at 4 °C. To enable equal protein loading in each lane, protein content was measured using a RC-PC protein assay kit (DC Protein Assay Kit, Bio-Rad, Hercules, CA) and then dissolved in Laemmli buffer.

Lysate proteins were separated by SDS-PAGE on 8.5% acrylamide gels and then electroblotted to PVDF membranes (Immobilon, Millipore, Bedford, MA). Blots were blocked with Odyssey Blocking Buffer (LI-COR Biosciences) following the manufacturer’s instructions and then incubated with primary (pendrin) antibody [17] overnight at 4 °C, followed by incubation for 2 h at room temperature with Alexa Fluor 680-linked anti-rabbit IgG (Invitrogen, A-21109, 1:5000). To correct for possible differences in lysate protein loading between lanes, membranes were Coomassie stained as reported previously [47]. Signals were visualized with an Odyssey Infrared Imaging System (LI-COR Biosciences). Immunoblot and Coomassie band densities were quantified using software program Image J (NIH, available at http://rsb.info.nih.gov/). Immunoblot band density was normalized to Coomassie gel band density in the region similar mobility. To confirm protein loading, actin immunoreactivity was quantified in the same blots using a rabbit, anti-actin antibody (Sigma Aldrich, #A2066, 1:1000).

### Statistics

Data are presented as the mean ± SE. Each “n” used in the statistical analysis represents data from separate animals. Statistical tests were performed with SigmaPlot 12.5 software. To test for statistical significance between two groups, an unpaired Student’s *t*-test was used. To compare multiple groups, one-way ANOVA was used with a Holm-Sidak post-test, as appropriate (see figure legends). The criterion for statistical significance was *P* < 0.05.

## Results

### α ENaC localizes to the perinuclear region of pendrin positive rat and mouse intercalated cells

**Fig. 1:**
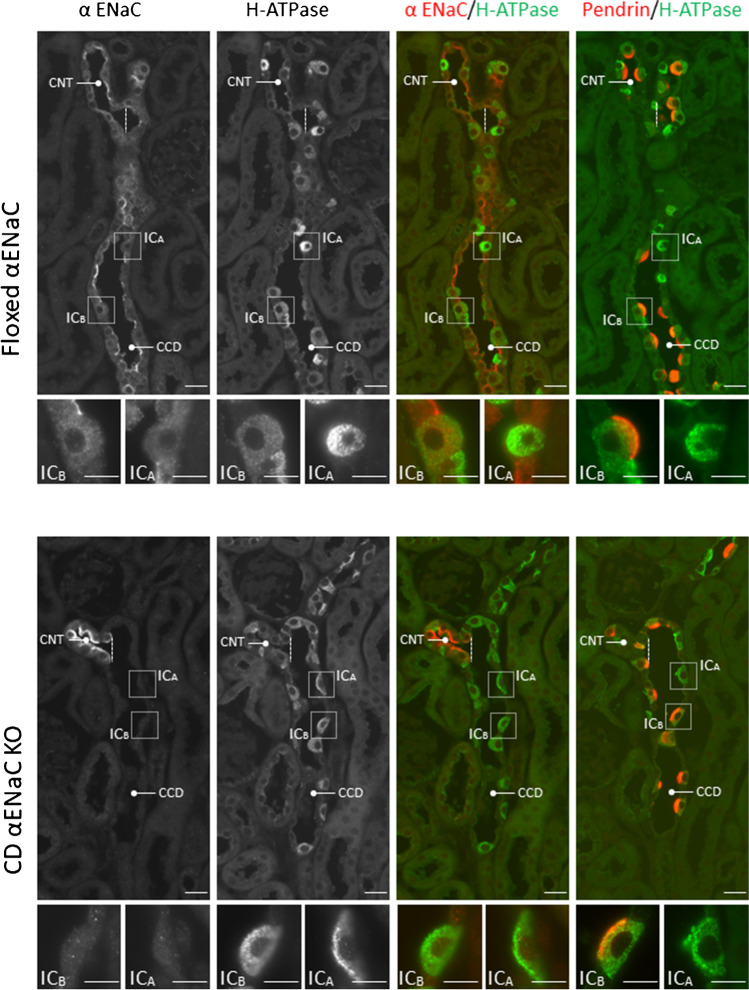
αENaC localizes to mouse intercalated cells. Consecutive sections of the kidneys from aldosterone-treated control (*Scnn1a*^loxlox^) and collecting duct-specific αENaC KO mice (HoxB7; *Scnn1α*^loxloxcre^) were co-immunostained for either αENaC and H^+^-ATPase or for pendrin and H^+^-ATPase. Squares (insets) show high magnifications of type A (IC_A_) and type B (IC_B_) intercalated cells in the collecting duct (CCD) and connecting tubule (CNT). Scale bars: 25 μm (overviews) and 10 μm (high magnifications). For simplicity, cells labeled as “IC_B_” represent both type B and non-A, non-B intercalated cells

Because *Scnn1a* transcript has been detected in ICs [4], we explored whether the protein encoded by this gene localizes to ICs, the IC subtype(s) that express this subunit and its subcellular distribution. To do so, we examined αENaC labeling in aldosterone-treated floxed αENaC mice (*Scnn1a*^loxlox^) and collecting duct-specific αENaC null mice (HoxB7: *Scnn1a*^loxloxcre^) [33] (Treatment #2, Fig. 1). To identify the cell types that express αENaC, consecutive sections were labeled either for αENaC and the B1 subunit of the H^+^-ATPase or for αENaC and pendrin. In both the floxed αENaC (*Scnn1a*^loxloxcre^) and in the collecting duct-specific αENaC null mice (HoxB7: *Scnn1a*^loxloxcre^), αENaC immunolabel was readily detected in the CNT. However, αENaC label was not detected in any cells within the CCD of the collecting duct αENaC KO mice (HoxB7: *Scnn1a*^loxloxcre^), which is consistent with the previous observation that αENaC is not expressed in the collecting duct of these mice [33].

**Fig. 2:**
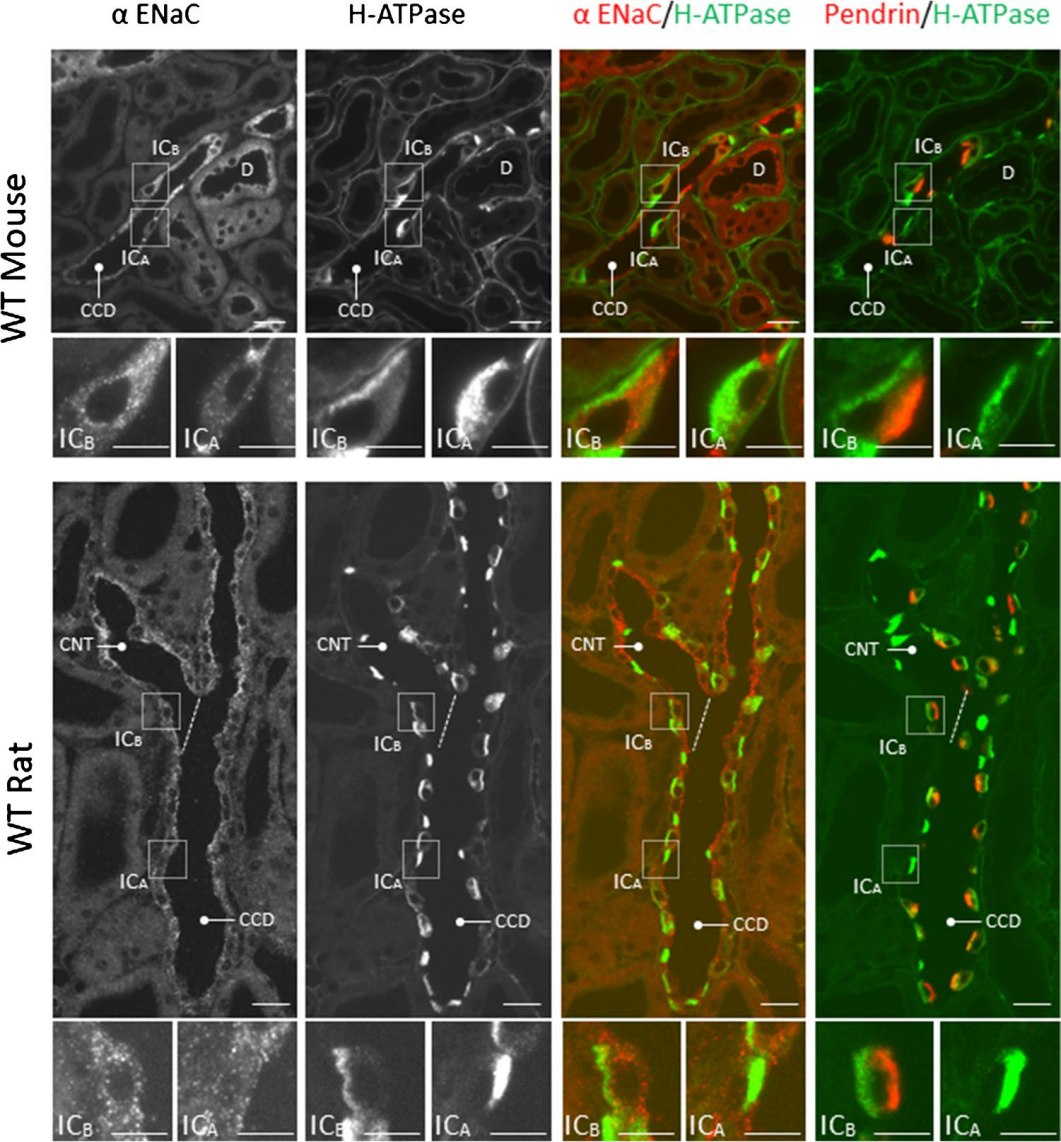
αENaC localizes to mouse and rat intercalated cells. Consecutive kidney cryosections from wild type (WT) rats and mice were co-immunostained for either αENaC and H^+^-ATPase or for pendrin and the H^+^-ATPase. Squares (insets) show type A (IC_A_) and type B (IC_B_) intercalated cells in the collecting duct (CCD) and connecting tubule (CNT) at high magnification. D, Distal convoluted tubule. Scale bars: 25 μm (overviews) and 10 μm (high magnification)

Further experiments explored the cell types that express αENaC in the mouse kidney (Fig. 1). In the control, floxed αENaC mice (*Scnn1a*^loxlox^), αENaC immunolabel was examined in 3 cell populations of the CCD. The first population did not express either the B1 subunit of the H^+^-ATPase or pendrin, which indicates they are principal cells. Many of these cells showed clear apical αENaC localization. The second cell population had distinct apical pendrin immunolabel and diffuse H^+^-ATPase label, consistent with either type B or non-A, non-B ICs, i.e. pendrin-positive ICs [14]. For simplicity, both pendrin-positive populations are labeled as type B ICs (Fig. 1). The third cell population was positive for the H^+^-ATPase, but negative for pendrin, which indicates type A ICs. In all intercalated cell populations, we observed diffuse, cytoplasmic αENaC immunolabel (inset). However, αENaC immunolabel was generally weak in the type A IC when compared with principal cells or type B ICs. Since type A IC immunostaining was often close to the limit of detection, we could not determine if αENaC is expressed at a significant level in any or in just a subset of these cells. Similar observations were made in kidney cryosections from wild type mice under basal conditions (Treatment #1, Fig. 2). We conclude that αENaC localizes to mouse pendrin-positive intercalated cells. Within these cells, αENaC has a diffuse, cytoplasmic distribution, which contrasts with the prominent apical label seen in principal or CNT cells.

Previous reports observed αENaC labeling in rat principal cells, but not in rat intercalated cells [12]. Therefore, we asked if the IC αENaC labeling we observed is unique to the mouse or if it is seen in other rodents. To answer this question, we explored αENaC localization in the rat kidney. As shown (Fig. 2), we observed αENaC in both principal cells and in pendrin-positive ICs of rat kidney, although αENaC was not readily detected in pendrin-negative inter-calated cells. We conclude that αENaC localizes to both principal cells and pendrin-positive intercalated cells in rat and mouse kidney.

### αENaC gene ablation does not change pendrin abundance or subcellular distribution

**Fig. 3:**
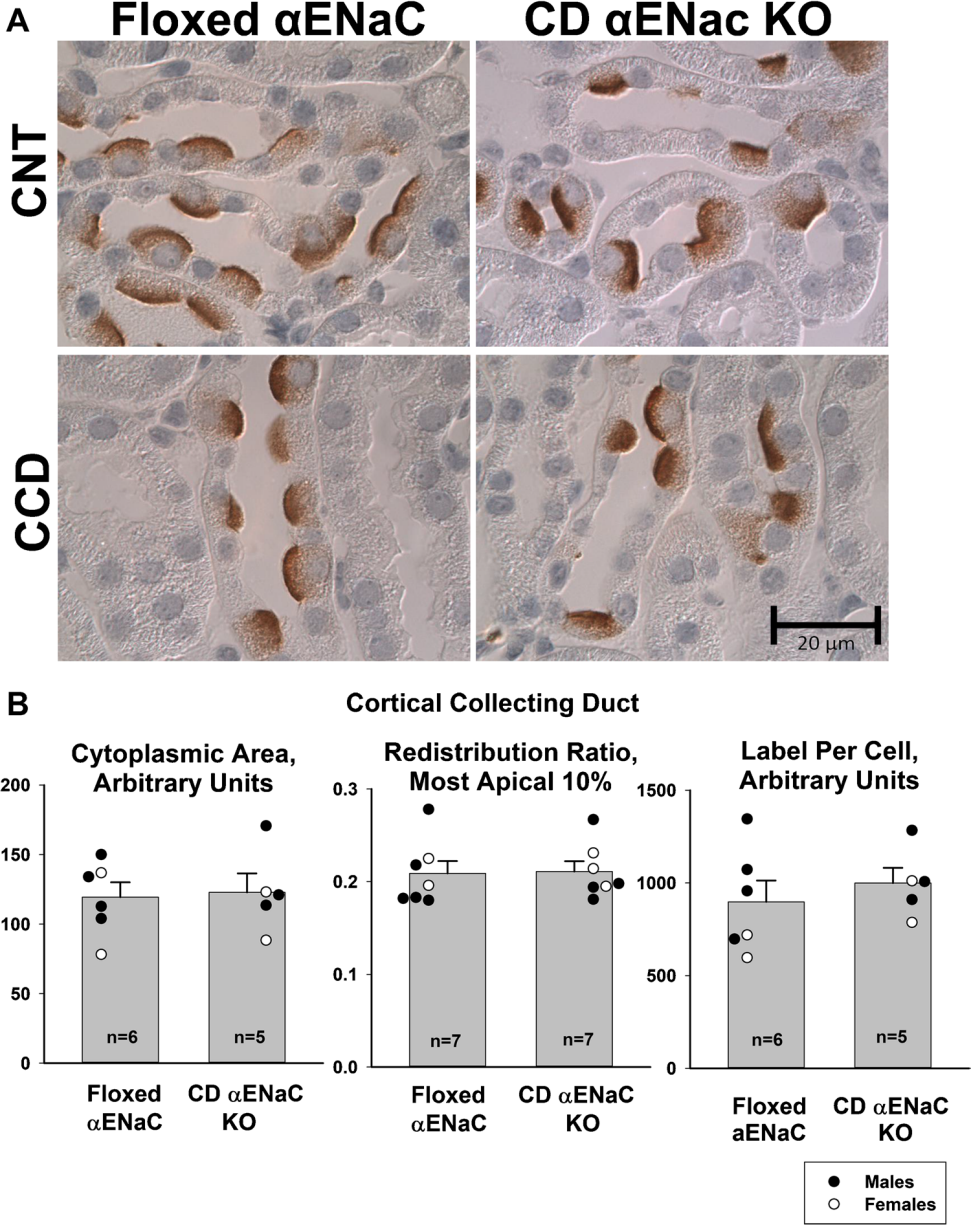
αENaC gene ablation does not change renal pendrin immunolabel intensity or subcellular distribution. Panel A shows pendrin immunolabel in a typical CCD and CNT from a male aldosterone-treated floxed αENaC and a male collecting duct-specific αENaC null mouse (HoxB7; *Scnn1a*^loxloxcre^, Treatment #2). Panel B shows cytoplasmic area of pendrin positive cells, pendrin label in the most apical 10% of the cell relative to total label (redistribution ratio) and the label per cell within the cytoplasm of pendrin positive cells of the CCD from both groups (Treatment #2). P = NS, unpaired Student’s *t*-test

Because αENaC localizes to pendrin-positive ICs and because aldosterone increases αENaC abundance in the kidney [20], we asked if αENaC gene ablation within intercalated cells and principal cells of the mouse CCD modulates pendrin label intensity or subcellular distribution in aldosterone-treated mice. Thus, we quantified pendrin label per cell as well as pendrin’s relative abundance in the most apical 10% of ICs in CCDs from age- and sex-matched controls (*Scnn1a*^loxlox^) and collecting duct specific αENaC null mice (HoxB7: *Scnn1a*^loxloxcre^) that received aldosterone for 7 days (Treatment #2). As shown (Fig. 3), both pendrin label per cell as well as pendrin’s relative abundance in the most apical 10% of the cell were similar in CCDs from aldosterone-treated floxed αENaC and in CD-specific αENaC null mice.

To exclude compensatory changes that might occur in the CNT following collecting duct αENaC gene ablation, we quantified pendrin abundance and subcellular distribution in the CNT of these aldosterone-treated collecting duct-specific αENaC KO (HoxB7: *Scnn1a*^loxloxcre^) and their floxed αENaC control littermates (*Scnn1a*^loxlox^, Fig. 3 and Table 1). As shown, pendrin immunolabel intensity and subcellular distribution were similar in CNTs taken from both groups**.** We conclude that while αENaC localizes to ICs. While collecting duct αENaC gene ablation markedly reduces Cl^−^ absorption in the mouse CCD [26], it does not significantly modulate pendrin abundance or subcellular distribution in aldosterone-treated mice.

**Table 1:** Pendrin label per cell and relative label in the most apical 10% in the CNT of aldosterone-treated floxed αENaC and collecting duct-specific αENaC null mice

	CNT	
	Cre ( −), floxed αENaC mice	Cre ( +), collecting duct-specific αENaC null
*n*	8	8
Cell height (arbitrary units)	1.76 ± 0.07	1.56 ± 0.13
Pendrin label intensity/cell (arbitrary units)	1366 ± 115	1243 ± 105
Pendrin abundance in the apical 10% relative to total abundance	0.363 ± 0.016	0.352 ± 0.009

There were 5 male and 3 female littermates in both the Cre ( −), floxed αENaC and the Cre ( +) collecting duct-specific αENaC groups all of which were on a C57Bl6/J background. Groups were compared with an unpaired Student’s *t*-test

### Constitutively increasing ENaC activity does not increase apical plasma membrane pendrin abundance

**Fig. 4:**
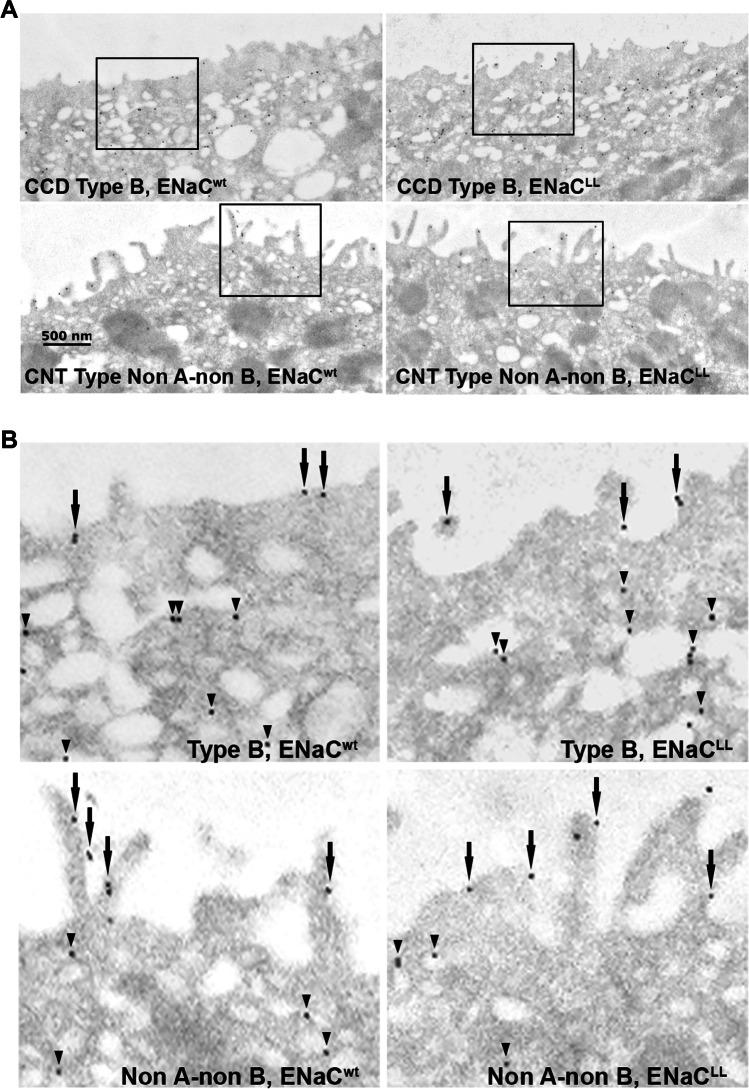
Constitutively upregulating ENaC does not increase apical plasma membrane pendrin immunolabel in NaCl-restricted mice. **A** Pendrin gold immunolabel is shown in a representative type B and a non-A, non-B intercalated cell taken from a wild type and a Liddle’s mouse following 7 days of the NaCl-restricted diet (Treatment #3). **B** Gold immunolabel is shown at higher magnification in the regions marked by the boxes (insets) in A. Arrows show immunogold on the apical plasma membrane, while arrowheads show gold label in the subapical space

We hypothesized that changes in principal or CNT cell ENaC activity might modulate pendrin abundance and/or function through a paracrine mechanism, independently of intercalated cell αENaC. To test this hypothesis, we employed mouse models of Liddle’s Syndrome, which have a truncation mutation in the cytoplasmic C terminus of the βENaC subunit that prevents channel endocytosis and degradation [30]. As a result, ENaC channel activity is greater in these mice than in wild type controls [30]. We therefore explored whether the increased ENaC activity seen in this mouse model amplifies either total or apical plasma membrane pendrin immunolabel. Because the Liddle’s sequence variant increases ENaC activity more following a NaCl-restricted than a NaCl-replete diet [5], we examined pendrin subcellular distribution and protein abundance by immunogold cytochemistry with morphometric analysis in age- and sex-matched wild type and Liddle’s mice following 7 days of moderate dietary NaCl restriction (Treatment #3). Figure 4 and Table 2 show that in both type B and non-A, non-B ICs, apical plasma membrane pendrin label per cell as well as total pendrin gold label per cell were similar in wild type and Liddle’s mice.

**Table 2:** Total and apical plasma membrane pendrin abundance in NaCl-restricted Liddle’s and wild type mice (Treatment #3)

	B cells		Non-A, non-B cells	
	Wild type	Liddle’s mice	Wild type	Liddle’s mice
*n*	4	4	4	4
Boundary length/cell (mm, × 10^–2^)	1.07 ± 0.12	1.23 ± 0.09	3.10 ± 0.20	4.00 ± 0.84
Apical plasma membrane Au/cell	6.96 ± 3.19	7.38 ± 3.69	39.7 ± 4.9	41.2 ± 4.3
Ratio of apical plasma membrane Au to cytoplasmic Au	0.0964 ± 0.0408	0.117 ± 0.025	1.55 ± 0.37	1.52 ± 0.204
Total Au/cell	73.8 ± 10.2	70.1 ± 3.3	68.7 ± 3.9	70.1 ± 9.4

Mice were on a C57Bl6/J background and studied following *Treatment #2*. Age and sex-matched wild type (ENaC^wt^) and Liddles (ENaC^LL^) littermates on a C57Bl6/J background were compared. The wild type group had 2 males and 2 females. The Liddle’s group had 1 male and 3 female littermates. Groups were compared with an unpaired Student’s *t*-test. Au, gold immunolabel

**Fig. 5:**
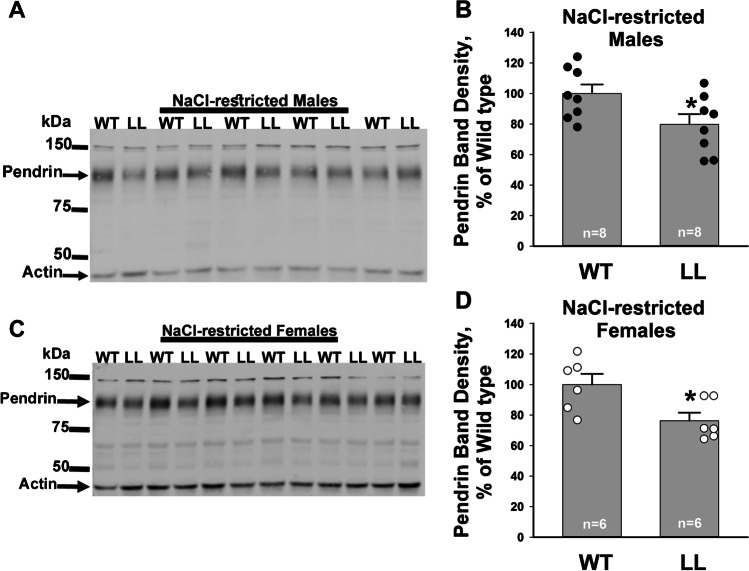
Constitutively upregulating ENaC does not increase pendrin total protein abundance in NaCl-restricted mice. Shown are typical immunoblots and their respective pendrin band density of kidney lysates from NaCl-restricted male (**A** and **B**) and female wild type (WT) and Liddle’s (LL) mice (**C** and **D**, Treatment #3). *P < 0.05, unpaired Student’s *t*-test

Further experiments examined pendrin protein abundance in kidney lysates from NaCl-restricted wild type and Liddle’s mice (Treatment #2). As shown (Fig. 5), in both male and female mice, pendrin abundance was the same or slightly lower in NaCl-restricted Liddle’s relative to wild type mice. We conclude that increasing ENaC channel activity through the Liddle’s mutation does not increase either total or apical plasma membrane pendrin abundance in NaCl-restricted mice.

### Constitutively increasing apical plasma membrane ENaC does not increase apical plasma membrane pendrin abundance when circulating aldosterone concentration is held constant

**Fig. 6:**
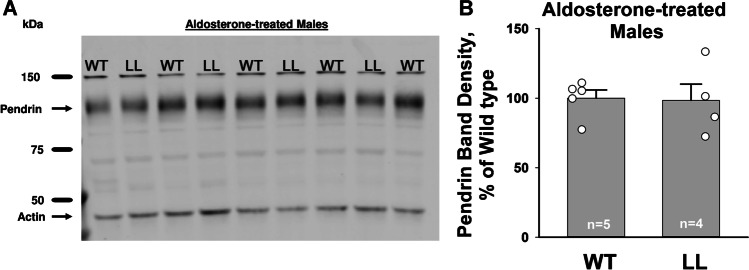
Constitutively upregulating ENaC does not increase pendrin protein abundance in kidneys from aldosterone-treated mice. Shown is an immunoblot (**A**) and its respective pendrin band density (**B**) of kidney lysates from aldosterone-treated male wild type (WT) and Liddle’s (LL) mice (Treatment #2). P = NS, unpaired Student’s *t*-test

While serum electrolytes and arterial blood gases were similar in the NaCl-restricted Liddle’s and wild type mice (Table 3), multiple previous studies reported lower aldosterone plasma concentration in NaCl-restricted Liddle than in their wild type controls [1, 29, 30]. Table 3 confirms this serum aldosterone concentration difference in female mice. Because aldosterone increases pendrin abundance and function, we hypothesized that that the Liddle’s mutation fails to increase pendrin abundance due to the lower circulating aldosterone concentration seen in the NaCl-restricted Liddle’s than wild type mice. To test this hypothesis, we examined pendrin abundance in Liddle’s and wild type mice following 10 days of an aldosterone infusion, where circulating aldosterone concentration is similar in both groups (Treatment #2, Table 3). Although ENaC channel activity is higher in the kidneys from the aldosterone-treated Liddle’s than wild type mice [27], Fig. 6 shows that pendrin band density is similar in aldosterone-treated male Liddle’s and wild type mice. We conclude that constitutively upregulating ENaC activity does not significantly increase pendrin total protein abundance.

**Table 3:** Serum aldosterone, electrolytes, and arterial blood gases in Liddle’s and wild type mice

	Arterial blood gases			Electrolytes		n		n
	pH	pCO_2_	cHCO_3_^−^	Na^+^	K^+^		Aldosterone	
		mm Hg	mM	mEq	mEq		nM	
*Female NaCl-restricted mice (Treatment #3)*								
Wild type	7.356 ± 0.010	46.9 ± 1.4	26.2 ± 0.4	145 ± 1	3.6 ± 0.1	6	3.37 ± 0.5	5
Liddle’s	7.388 ± 0.016	43.2 ± 1.9	25.9 ± 0.9	144 ± 1	3.5 ± 0.1	6	2.53 ± 0.14	5
*Male NaCl-restricted mice (Treatment #3)*								
Wild type	7.384 ± 0.005*	46.5 ± 2.3	27.8 ± 1.3	143 ± 1	3.4 ± 0.1	5	NM	
Liddle’s	7.424 ± 0.006*	42.3 ± 1.5	27.8 ± 1.2	143 ± 1	3.3 ± 0.1	5	NM	
*Male aldosterone-treated mice (Treatment #2)*								
Wild type	7.574 ± 0.012	44.9 ± 3.4	41.1 ± 1.3^σ^	146 ± 1	2.5 ± 0.2	5	10.6 ± 0.7	5
Pendrin KO	7.627 ± 0.012	49.3 ± 1.3	51.5 ± 0.2^σ^	146 ± 1	2.6 ± 0.3	3		
Liddle’s	7.519 ± 0.012^τ^	47.0 ± 0.9	38.2 ± 0.7^ρ^	148 ± 1	2.0 ± 0.1	4	12.9 ± 1.9	4
Liddle’s/pendrin KO	7.694 ± 0.040^τ^	54.5 ± 7.1	59.6 ± 3.5^ρ^	147 ± 2	2.3 ± 0.1	4		

cHCO_3_^−^, calculated HCO_3_^−^ concentration. Aldosterone-treated mice were compared using a one-way ANOVA with a Holm-Sidak post-test. ρ, σ and τ indicate values that were statistically different (*P* < 0.05). NaCl-restricted male and female mice were compared separately using an unpaired Student’s t-test. * indicates values that were statistically different (*P* < 0.05). Mice were on a 129SvEv background; *NM*, not measured

### Constitutively upregulating ENaC does not significantly increase pendrin-dependent Cl^−^ absorption in aldosterone-treated mice

**Fig. 7:**
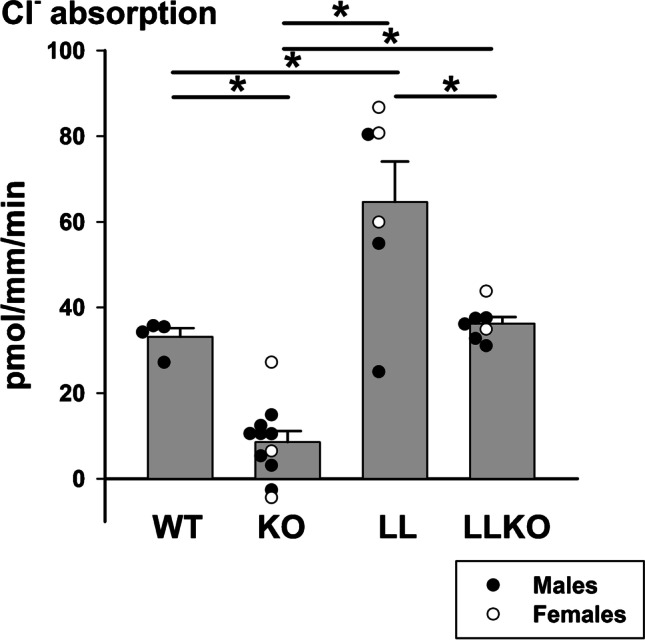
Constitutively increasing ENaC activity does not significantly increase pendrin-dependent Cl^−^ absorption in mouse CCD from aldosterone-treated mice. Cl^−^ absorption, J_Cl_, was measured in CCDs taken from mice given aldosterone for 5–7 days (Treatment #2) that were homozygous Liddle’s and homozygous pendrin null (ENaC^LL^; *Slc26a4*^−/−^ or LLKO, *n* = 7), homozygous for both Liddle’s and wild type pendrin (ENaC^LL^; *Slc26a4* ^+/+^ or LL, *n* = 6), homozygous wild type ENaC and homozygous pendrin null (ENaC^WTWT^; *Slc26a4*^*−/−*^ or KO, *n* = 11) and mice homozygous for wild ENaC and pendrin (ENaC^WTWT^; *Slc26a4*^+*/*+^ or WT, *n* = 4). *P < 0.05, by one-way ANOVA with a Holm-Sidak post-test

Further experiments explored whether constitutively upregulating ENaC activity changes the pendrin-dependent component of Cl^−^ absorption in mouse CCD. To answer this question, we measured Cl^−^ absorption (J_Cl_) in CCDs from age- and sex-matched aldosterone-treated pendrin null and wild type pendrin mice (Treatment #2) that expressed either wild type ENaC (ENaC^WT/WT^; *Slc26a4*^+*/*+^*,* WT or ENaC ^WT/WT^; *Scl26a4*^*−/−*^, KO) or that were homozygous for the Liddle’s mutation (ENaC ^L/L^; *Slc26a4* ^+*/*+^, LL or ENaC^L/L^; *Slc26a4*^*−/−*^ or LLKO). Figure 7 shows that under these conditions, Cl^−^ absorption is greater in the Liddle’s than in wild type mice. However, pendrin gene ablation produced a similar change in Cl^−^ absorption, J_Cl,_ in CCDs from both Liddle’s mice and in mice that express wild type ENaC. As shown (Fig. 7), Cl^−^ absorption was 28.4 pmol/mm/min lower in CCDs from aldosterone-treated Liddle’s mice that were pendrin null relative to those with wild type pendrin. Similarly, in mice that express wild type ENaC, Cl^−^ absorption was 24.6 pmol/mm/min lower in CCDs from aldosterone-treated pendrin KO than pendrin wild type mice. Therefore, the Liddle’s variant did not significantly magnify the fall in Cl^−^ absorption seen with pendrin gene ablation, despite the higher ENaC channel activity observed in Liddle’s than in wild type mice measured under the same treatment conditions [27]. We conclude that in aldosterone-treated mice, stimulating ENaC channel activity does not significantly increase the pendrin-dependent component of Cl^−^ absorption.

## Discussion

This study demonstrates that αENaC localizes to not only to principal cells but also to pendrin-positive intercalated cells of the rat and mouse kidney. αENaC label was clearly detected in pendrin-positive intercalated cells, although less intense than in either principal cells or in CNT cells. However, in pendrin-negative, type A ICs, αENaC immunolabel was either very low or absent. Our observations at the protein level nicely match previous observations made with single cell mRNA sequencing. In mouse [4, 31] and human [21] kidney, single cell RNAseq studies demonstrated αENaC mRNA expression in pendrin-positive ICs, although αENaC expression was much lower, or absent, in pendrin-negative (type A) ICs. While these RNAseq studies showed clear αENaC mRNA expression in both principal and in pendrin-positive intercalated cells, β and γENaC subunit mRNA expression was detected only in principal cells. As such, this study examined the expression of the α, but not the β or γ, ENaC subunits in pendrin-positive intercalated cells. Since apical ENaC surface targeting requires the expression of all three ENaC subunits within that cell [8], the diffuse αENaC immunolabel observed in pendrin positive ICs may reflect the absence of the β and γ ENaC subunits in these cells. This diffuse αENaC label contrasts with the strong apical pendrin immunolabel observed and makes it unlikely that these proteins directly associate within intercalated cells.

The presence of αENaC label in pendrin-positive intercalated cells from wild type mice, but not in collecting duct αENaC KO mice, confirms the specificity of the αENaC labeling observed in this study. Because an αENaC KO rat is unavailable, we cannot confirm the specificity of the αENaC immunolabel with similar experiments in rat kidney. However, since the distribution of αENaC labeling within rat and mouse kidney is similar, it is unlikely that the rat αENaC immunolabel is nonspecific. Moreover, single-cell RNAseq data detected αENaC mRNA expression in rat intercalated cells, although the IC subtype that expresses this αENaC mRNA was not determined [6].

Previous studies [12, 20], including our own [19], observed α, β and γENaC subunit expression in principal cells and CNT cells, but not within intercalated cells. We cannot explain why the present study, but not these earlier reports, observed αENaC immunolabel within ICs. The anti-body used in this study may be more sensitive than those used previously and therefore detects αENaC at lower levels of expression. However, other explanations are also possible.

Based on the αENaC labeling we observed within ICs, we hypothesized that intercalated cell αENaC contributes to ion transport within intercalated cells, independently of the β and γNaC subunits. When αENaC is expressed in oocytes, a small amiloride-sensitive current is observed, although current is much greater when the β and γ subunits are co-expressed [3]. Moreover, αENaC and the acid-sensing ion channel 1a (ASIC1a) associate within secretory, alveolar type 2 pneumocytes (AT2), independently of the β and γENaC subunits, to generate a small benzamil-insensitive Na^+^ current [38]. This channel has a larger conductance, a shorter mean open and closed time, and a much lower amiloride sensitivity than does ENaC [38]. Unlike ENaC, this channel does not show selectivity for Na^+^ over K^+^ [38]. Therefore, while αENaC may mediate some channel activity within intercalated cells, independently of the β and γ ENaC subunits, it is likely very low. Significant αENaC-mediated transepithelial ion transport is also unlikely due to the absence of clear apical αENaC immunolabel.

Although intercalated cell αENaC does not likely mediate significant transepithelial ion transport, αENaC gene ablation within both principal cells and intercalated cells of CCDs from aldosterone-treated mice markedly reduced Cl^−^ absorption and transepithelial voltage, *V*_T_ [26] as well as ENaC channel activity [33], although Na^+^ and K^+^ balance, as well as serum aldosterone, are unchanged [33]. Therefore, the absence of an effect of αENaC gene ablation on pendrin abundance or subcellular distribution is not readily explained by a systemic, indirect effect of collecting duct αENaC gene ablation that blunts ENaC-dependent changes in pendrin abundance or function. Moreover, constitutively increasing ENaC activity paradoxically reduces pendrin total protein abundance in NaCl-restricted mice. Why pendrin abundance is lower in the NaCl-restricted Liddle’s relative to wild type controls is not known but may be from the lower serum aldosterone concentration expected in the NaCl-restricted Liddle’s relative to wild type mice [1, 29, 30].

Collecting duct αENaC gene ablation most likely reduces Cl^−^ absorption in the mouse CCD through a pendrin-independent mechanism. The rodent CCD absorbs Na^+^ and Cl^−^ through an electrogenic, benzamil-sensitive and an electroneutral, thiazide-sensitive mechanism [18, 37]. The benzamil-insensitive component of Na^+^ and Cl^−^ absorption is mediated, at least in part, by the apical Na^+^-independent, Cl^−^/HCO_3_^−^ exchanger, pendrin, encoded by *Slc26a4*, and the Na^+^-dependent, Cl^−^/HCO_3_^−^ exchanger, NDCBE, encoded by *Slc4a8,* which act in tandem [18]. Benzamil-sensitive NaCl absorption occurs through ENaC-mediated Na^+^ absorption, which provides the driving force for paracellular Cl^−^ absorption and for transcellular, electrogenic Cl^−^ transport. Because αENaC gene ablation markedly reduces both the total and the benzamil-sensitive component of Cl^−^ absorption in CCDs from aldosterone-sensitive mice [26], while pendrin abundance and subcellular distribution were unchanged, the lower Cl^−^ absorption and *V*_T_ seen in these KO mice likely occurs from changes in electrogenic Cl^−^ transport, rather than electroneutral, pendrin-dependent mechanisms. However, we cannot exclude the possibility that eliminating intercalated cell αENaC reduces Cl^−^ absorption in the CCD by affecting another intercalated cell transporter or exchanger, which raises intercalated cell intracellular Cl^−^ concentration or reduces HCO_3_^−^ concentration and thereby reduces the driving force for pendrin-mediated Cl^−^/HCO_3_^−^ exchange.

We used mouse models of Liddle’s syndrome to explore the possibility that ENaC channel activity within CNT and principal cells modulates pendrin abundance or function. While the Liddle’s sequence variant raised both ENaC channel activity as well as total Cl^−^ absorption in the CCD of aldosterone-treated mice, it did not significantly change the pendrin-dependent component of Cl^−^ absorption, nor did it increase pendrin total protein abundance. Therefore, ENaC channel activity modulates Cl^−^ absorption in the mouse CCD through a mechanism that occurs, at least in part, independently of pendrin. Most likely, ENaC activity modulates Cl^−^ absorption by increasing the lumen-negative transepithelial voltage, which enhances electrogenic, benzamil/amiloride-sensitive transepithelial Cl^−^ absorption and does not appreciably change the pendrin-dependent component of Cl^−^ absorption.

The present and previous studies show that αENaC gene ablation reduces [26], while increasing ENaC activity increases Cl^−^ absorption in the mouse CCD. However, while ENaC changes Cl^−^ absorption, it does so without changing pendrin abundance, subcellular distribution, or function. These results are in contrast with our previous observations that pendrin gene ablation reduces total and apical ENaC subunit abundance as well as ENaC channel open probability and ENaC channel activity under the same treatment conditions (Treatments #2 & #3) [15, 24, 27].

We conclude that αENaC and pendrin co-localize to type B and non-A, non-B ICs in rat and mouse kidney, although the physiological role of αENaC within intercalated cells, remains to be determined. ENaC modulates Cl^−^ absorption in the mouse CCD by changing the benzamil-sensitive component of Cl^−^ absorption, independently of pendrin. While pendrin markedly regulates ENaC abundance, subcellular distribution, and function either in NaCl-restricted or in aldosterone-treated mice, ENaC does not have a similar effect on pendrin under the same treatment conditions.
